# Acupuncture Alleviates Menstrual Pain in Rat Model via Suppressing Eotaxin/CCR3 Axis to Weak EOS-MC Activation

**DOI:** 10.1155/2022/4571981

**Published:** 2022-01-13

**Authors:** Wen-Yan Yu, Liang-Xiao Ma, Yuan Tian, Jie-Dan Mu, Zhou Zhang, Tian-Yi Sun, Xu Qian, Jun-Xiang Wang

**Affiliations:** ^1^School of Acupuncture-Moxibustion and Tuina, Beijing University of Chinese Medicine, Beijing 100029, China; ^2^The Key Unit of State Administration of Traditional Chinese Medicine, Evaluation of Characteristic Acupuncture Therapy, Beijing 100029, China; ^3^School of Nursing, Beijing University of Chinese Medicine, Beijing 100029, China

## Abstract

**Introduction:**

Emerging data show that chemokine-mediated inflammation is involved in the occurrence and maintenance of pain. Recent evidence suggests that eotaxin levels rise when dysmenorrhea happens. The purpose of this study is to investigate whether eotaxin/CC chemokine receptor 3 (CCR3) axis, a key regulatory pathway for eosinophils (EOS) recruitment, is involved in acupuncture analgesia for dysmenorrhea.

**Methods:**

After the cold congealing dysmenorrhea (CCD) rat model prepared, animals received perpendicular needling (PN) and transverse needling (TN) at SP6, respectively, for 20 min. The CCR3 agonist CCL11 was administered 30 min prior to acupuncture. Pain behavior was assessed via a writhing response. The uterine contraction test was detected by an electrophysiological method. Eotaxin, histamine (HIS), and interleukin-6 (IL-6) levels were evaluated by ELISA. The expression of CCR3 and histamine H1 receptor (H1R) was analyzed by RT-qPCR and Western blot. The expression of EOS, mast cells (MCs), eosinophil peroxidase (EPO), and eosinophil cationic protein (ECP) was assessed by hematoxylin-eosin staining (HE), Toluidine Blue staining (TB), and immunohistochemistry, respectively.

**Results:**

Acupuncture prominently attenuated the menstrual pain in CCD rats, particularly TN technique. Electrophysiological recording data showed that the increased uterine contractility was ameliorated by acupuncture. In addition, TN decreased the release of eotaxin, HIS, IL-6, and the expression of CCR3 and H1R. HE, TB staining, and immunohistochemistry experiments showed that the increased expression of EOS, MCs, EPO, and ECP in uterine tissues was reversed by TN. Furthermore, we found that the effects of TN against CCD-induced menstrual pain, increased ECP expression, and HIS level were abolished by CCL11.

**Conclusion:**

TN alleviated menstrual pain by improving the uterine inflammatory environment via suppressing eotaxin/CCR3 axis to weak EOS-MC activation in CCD rats. The study findings support the acupuncture as a promising approach for dysmenorrhea, meanwhile, indicating the importance of performing appropriate needling technique.

## 1. Introduction

As a common gynecological disorder, primary dysmenorrhea (PD) refers to spasmodic pain in the lower abdomen without obvious pelvic organic lesions before, during, or after menstruation [1]. About 45% to 90% of women in the world suffer from dysmenorrhea, among which 10% to 25% are severe dysmenorrhea [2]. PD leads to not only physical pain to patients but also huge economic loss to the society [3]. At present, PD is commonly treated with nonsteroidal anti-inflammatory medications and oral contraceptives. However, these drugs are accompanied by some side effects, such as headache, dizziness, fatigue, loss of appetite, nausea, vomiting, and drowsiness [4], and approximately 18% of women do not respond these [5]. Therefore, prevention and treatment of PD are important.

The menstruation could be regarded as an inflammatory event [6]. Previous researches suggested that eosinophils (EOS) could be seen in endometrium before and during menstruation [7, 8]. EOS and MCs played essential roles in morphological changes associated with the menstrual cycle and were believed to be important inflammatory cells involved in uterine inflammatory microenvironment [7, 9]. Recent evidence showed that chemokine-mediated inflammation involved in the introduction and maintenance of pain, and chemokines revealed as new players in pain control [10, 11]. Eotaxin, the most selective chemoattractant protein for EOS [12], can trigger the EOS recruitment into the inflammatory tissues via CC chemokine receptor 3 (CCR3), which is expressed in EOS [13, 14]. A recent study has showed that the serum level of eotaxin is increased in patients with primary dysmenorrhea [15]. Eosinophil cationic protein (ECP) has been shown to stimulate mast cells (MCs) to release histamine (HIS), which promotes uterine contractions and exacerbates pain [16]. Therefore, inactivation of eotaxin/CCR3 axis weakening EOS-MC activation could be involved in anti-inflammatory and analgesic mechanism subsequently modulating menstrual pain.

As a nonpharmacologic intervention, acupuncture has been increasingly used for treatment of pain [17], including PD [18, 19], and is recognized as an effective and safe therapy with little risk of serious adverse effects [20]. Recently, it has been pyramidally recognized that the anti-inflammatory effect of acupuncture contributes to its analgesic mechanism [21–23]. Moreover, acupuncture showed positive results of relieving asthma and allergic rhinitis via reducing eotaxin secretion [24, 25]. Sanyinjiao (SP6), located on the tibial aspect of the leg, posterior to the medial border of the tibia and 3 B-cun (proportional bone cun), above the prominence of the medial malleolus [26], is the intersection point of liver, spleen, and kidney channels, which is closely related to the uterus with the effects of moving Qi and regulating blood. A variety of studies affirmed that SP6 is one of the most common used points for PD, with satisfactory analgesic effect [27, 28]. Clinically, needling techniques, such as the depth, angle, and direction of needling, play an essential role for acupuncture efficacy. However, the mechanism induced by different needling techniques at the same acupuncture point is often overlooked and worthy of further study. Our previous studies provided evidence that needling techniques could affect the pain-relief effect of SP6 for PD via different pathways [29–31].

Considering that there are data showing the modulation of eotaxin on menstrual pain, we hypothesized that acupuncture might reduce eotaxin and CCR3, thus weakening the EOS-MC activation to alleviate the inflammatory environment and, subsequently, relieving menstrual pain.

## 2. Materials and Methods

### 2.1. Animals

Three-month-aged female Sprague-Dawley (SD) rats of sexually mature and nonmating weighing 230 ± 20 g were obtained from Beijing Vital River Laboratory Animal Technology. All rats were caged individually and handled in a constant environment with food and water freely available throughout the experiment. The constant environment was maintained at 23 ± 2°C with humidity of 45% ± 5% and a standard 12-hour light-and-dark cycle (lights on at 08:00 am). Animal care and experiments were performed strictly in conformity with the Guidelines for the Care and Use of Laboratory Animals by the National Institutes of Health and approved by the Animal Ethics Committee of Beijing University of Chinese Medicine (Approval No. BUCM-4-2019070502-3015), and all efforts were made to minimize animal suffering.

### 2.2. Establishment of Cold Congealing Dysmenorrhea (CCD) Rat Model

**Figure 1:**
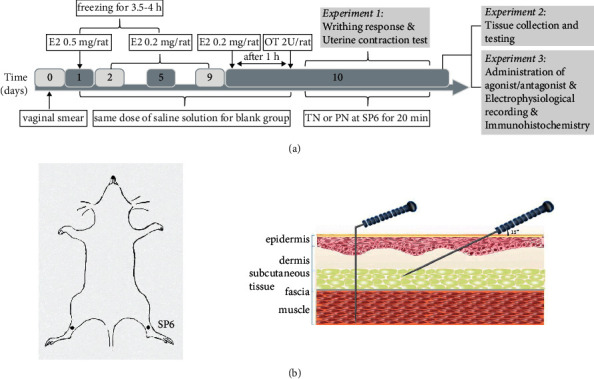
(a) Experimental design. (b) The location of SP6 and acupuncture interventions. Perpendicular needling method, PN; transverse needling, TN.

The CCD rat model was established as described previously [5, 32, 33]. The rats were frozen and subcutaneously injected with Estradiol Benzoate (Ningbo Sansheng Biotechnology Co., Ltd., Ningbo, China) in the abdomen [34, 35]. First, female rats were selected based on the estrous cycle for the induction of dysmenorrhea. The vaginal smear was collected and analyzed with a light microscope, and the selected rats during diestrus were subcutaneously injected Estradiol Benzoate for 10 days (0.5 mg/rat on the 1st and 10th days, and 0.2 mg/rat for the 2nd to 9th days). Second, on the 1st to 5th days after administration, the rats were placed in a −25°C freezer for continuous freezing for 4 h and ventilated for 5 s at 2 h. Third, 1 h after the last administration on the 10th day, each rat was injected 2U of oxytocin (Chifeng Bone Pharmaceutical Co., Ltd., Chifeng, China) intraperitoneally. The blank group was treated with the same dose of saline solution (Figure 1(a)).

### 2.3. Experimental Design

#### 2.3.1. Experiment 1

To observe the therapeutic effect of acupuncture and to find the most effective needling method, we performed the writhing response and the uterine contraction test to evaluate menstrual pain. The rats were divided randomly into 4 groups: the blank group, the CCD model group, the Model + perpendicular needling (PN) group, and the Model + transverse needling (TN) group (*n* = 12/group). The writhing response (*n* = 6/group) and the uterine contraction test (*n* = 6/group) were evaluated.

#### 2.3.2. Experiment 2

To investigate whether TN-induced analgesia associated with the eotaxin/CCR3 pathway, we detected the level of the key factors and the expression of inflammatory cells. The rats were divided randomly into 3 groups: Blank, Model, and Model + TN (*n* = 6/group).

#### 2.3.3. Experiment 3

To explore the role of CCR3 played in the mechanism of TN, intraperitoneal injection of CCR3 selective antagonist SB328437 and agonist CCL11 was performed. The rats were randomly divided into the following 6 groups: Model, Model + SB328437, Model + Vehicle1, Model + CCL11 + TN, Model + Vehicle2 + TN, and Model + Vehicle1 + TN (*n* = 6/group). SB328537 and CCL11 were dissolved in Vehicle 1 or Vehicle 2, respectively.

### 2.4. Acupuncture Interventions

After oxytocin injection on the 10th day, Sanyinjiao (SP6), located 10 mm proximal to the prominence of the medial malleolus [34, 35], was needled bilaterally by an ameliorated filiform needle (Φ0.18 mm × 5 mm) in 2 needling groups. In the Model + PN group, needles were inserted vertically at a depth of 4-5 mm. In the Model + TN group, needles were inserted transversely 4-5 mm toward the abdomen, at an angle of about 15° between the needle and the skin surface (Figure 1(b)). In order to observe the writhing response effectively, we ameliorated the needle as shown in Figure 1(b). The needle handle was bent and then fixed with an adhesive tape after a needle insertion; therefore, the rats could move freely during needle retention. Needles were retained for 20 min. Both the blank group and the model group used the same method for grasping fixation, but no treatment was performed.

### 2.5. Drug Administration

The CCR3 receptor antagonist SB328437 (ab120648, Abcam, Cambridge, UK), dissolved in DMSO (Vehicle1), was administered intraperitoneally at a dose of 5 mg/kg 30 min prior to the acupuncture. The specific CCR3 agonist CCL11 (420-ME-100/CF, R&D Systems, MN, USA), which was reconstituted at 100 *μ*g/mL in sterile PBS (Vehicle2), was injected intraperitoneally at a dose of 10 *μ*g/per animal 30 min before the acupuncture. The dosage and route of drug administration were based on the previous study with certain minor modifications according to the experimental condition [36].

### 2.6. Behavioral Test

#### 2.6.1. Writhing Response

The writhing response of rats was observed within 3 or 20 min after injection of oxytocin on the 10th day. The latency and score of the writhing response, which were proposed by Schmauss C, were recorded [37].

### 2.7. Electrophysiological Recordings In Vivo

The uterine contraction test was recorded by the BL420S Biological Function Experimental System Recorder (Chengdu Taimeng Technology Co., Ltd., Chengdu, China). After injection of Estradiol Benzoate on the 10th day, intramuscular injection of 1% pentobarbital sodium (40 mg/kg) was performed in the rats. And a 2-3 cm longitudinal incision was performed at about 0.5 cm apart from the midline of the abdomen. The line was threaded at 1 cm on the bifurcation of uterine horn, and the free end of the line was connected to the tension sensor. After 0.1 g of afterload was settled, the uterine contraction wave was recorded. 2U oxytocin was injected directly into the uterus in the model group and 2 needling groups, and saline in the same dose injected in the blank group. Results were recorded 20 min after acupuncture intervention in 2 needling groups: uterine activity = number of uterine contraction wave × peak-to-peak wave value.

### 2.8. ELISA

According to the manufacturer's instructions, Rat Eotaxin/CCL11 ELISA Kit (RGB-60563R, RGB & CHN, Beijing, China), Rat HIS ELISA Kit (RGB-60351R, RGB & CHN), and Rat IL6 ELISA Kit (RGB-60302R, RGB & CHN) were used for the measurement of eotaxin, histamine, and IL6. After anesthesia, the blood and uterus were obtained. Blood was immediately placed in a biopsy tube with EDTA to prevent clotting. Then the whole blood was centrifuged to separate the plasma. The plasma samples were stored in the −80°C until assays were performed. An appropriate amount of uterine tissue was cut and weighed, and a certain amount of PBS was added (tissue mass : PBS = 1 : 20). After homogenate with a homogenizer (Lanyi-GTM, Shanghai, China), centrifugate for about 10 minutes (4000 r/min), collect the supernatant, and then detect the total protein. Protein concentration in tissue extract = detected protein concentration in the extract/TP value of the extract.

### 2.9. Real-Time Quantitative Polymerase Chain Reaction (RT-qPCR)

Total RNA of the cells was isolated with TRIzol (Invitrogen) and then was reversely transcribed into cDNA using a Prime-Script RT reagent kit (CW2569, Kangwei Century, Beijing, China). The gene expression levels of CCR3 and histamine H1 receptor (H1R) were quantified by SYBR FAST qPCR Kit Master Mix (2×) (KK4601, KAPA Biosystems, MA, USA) and detected by the StepOne Plus Real-Time PCR Detection System (ABI, CA, USA). The PCR reaction conditions were as follows: predenaturation at 95°C for 3 minutes, denaturation at 95°C for 10 s, annealing/extension at 59°C for 60 s, and a total of 40 cycles of denaturation to extension. The relative expression levels of CCR3 mRNA and H1R mRNA were calculated by the 2^−△△Ct^ method as normalized by *β*-actin (an endogenous housekeeping gene). Primer sequences and amplification sizes are presented in Table 1.

### 2.10. Western Blot

The uterine tissue was homogenized in radioimmunoprecipitation assay buffer. And then the homogenate was centrifuged at 13,000 rpm for 20 min at 4°C and the supernatant was collected. The protein concentration was determined using the bicinchoninic acid method (BCA) according to the kit's instruction (02912E, Kangwei Century, Beijing, China), and 20 mg of protein was loaded in each lane. Protein samples were separated on 5 to 10% sodium dodecyl sulfate (SDS)-polyacrylamide gel electrophoresis (PAGE) gels and electrophoretically transferred to polyvinyl difluoride (PVDF) membranes (Millipore, MO, USA). The membranes were blocked with 5% nonfat milk at room temperature for 1 hour, followed by overnight incubation at 4°C with the following primary antibodies diluted in blocking buffer : anti-CCR3 (1 : 1000, ab32512, Abcam) and anti-HR1R (1 : 500, BS-6663R, Bioss, Beijing, China). Subsequently, the immunoblots were incubated with second antibodies (1 : 10000, 111-035-003, Jackson, PA, USA) for 1 hour at room temperature. GAPDH (1 : 1000, 5174, CST, MA, USA) was used as an internal control. The immunoreactivity was detected by an ECL chemiluminescence detection system (WBKLS0500, Millipore) and analyzed by an image analysis system (Tanon 4200, Shanghai, China).

### 2.11. Histological Analysis

The uterine tissue was immersed in 4% paraformaldehyde for more than 24 h. After dehydration, tissues were imbedded in paraffin and sliced to a thickness of 4–6 *μ*m. Then, hematoxylin-eosin staining (HE) or Toluidine Blue Staining (TB) were, respectively, used to analyze the EOS and MCs. The sliced tissues were stained with hematoxylin and eosin or toluidine blue. The slides were examined at 800x or 400x or 200x magnification with a microscope.

### 2.12. Immunohistochemistry

The embedded uterine tissue slides (4–6 *μ*m) were initially treated for deparaffinization, rehydration, and antigen retrieval, followed by 3% H_2_O_2_ incubation for 30 minutes. Sections were incubated with anti-EPX (1 : 200, BS-3881R, Bioss) and anti-ECP (1 : 200, BS-1754R, Bioss) and then incubated with peroxidase-labeled IgG secondary antibody (PV-6000, Beijing ZSGB Biotechnology Co., Ltd., Beijing, China). Fields from each slide were examined and photographed under a light microscopy (×400). Image-Pro Plus 6.0 image analysis software (media controls, Silver Spring, MD, USA) was used for analyzing the values of total cross-sectional integrated optical density (IOD) of each image.

### 2.13. Statistical Management

Data were expressed as mean ± SEM. All data were normally distributed and one-way ANOVA was carried out. Homogeneity of group variances was checked with a Levene's test. The Bonferroni test was used to evaluate homogeneously distributed data, and the Tamhane T2 test was used to evaluate nonhomogeneous data. The value of *P* < 0.05 was considered statistically significant. SPSS 23.0 software was used for the statistical analysis.

## 3. Results

### 3.1. Acupuncture Alleviated Menstrual Pain in CCD Rats

**Figure 2:**
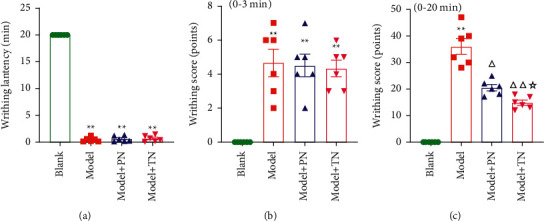
Acupuncture alleviated menstrual pain in cold congealing dysmenorrhea (CCD) rats. (a) The writhing latency. (b) The writhing scores in 3 min. (c) The writhing scores in 20 min. Data were analyzed by the Tamhane T2 post hoc test and expressed as means ± SEM (*n* = 6 rats/group). ^*∗∗*^*P* < 0.01 vs. Blank group; ^△△^*P* < 0.01 vs. Model group; ^△^*P* < 0.05 vs. Model group;^☆^*P* < 0.05 vs. Model + PN group.

As shown in Figure 2, the writhing latencies (Figure 2(a)) and writhing scores in 3 min (Figure 2(b)) of the rats in each model group were significantly different from those of the rats in the Blank group (*P* < 0.01), which indicated that the dysmenorrhea model was successfully established.

After acupuncture, compared with model group, a writhing score in 20 min significantly decreased in the two acupuncture groups (*P* < 0.05, *P* < 0.01, Figure 2(c)). Interestingly, a writhing score in 20 min significantly decreased in the Model + TN group compared with the Model + PN group (*P* < 0.05, Figure 2(c)), indicating that TN performs better in dysmenorrhea.

### 3.2. Acupuncture Attenuated Uterine Contraction in CCD Rats

**Figure 3:**
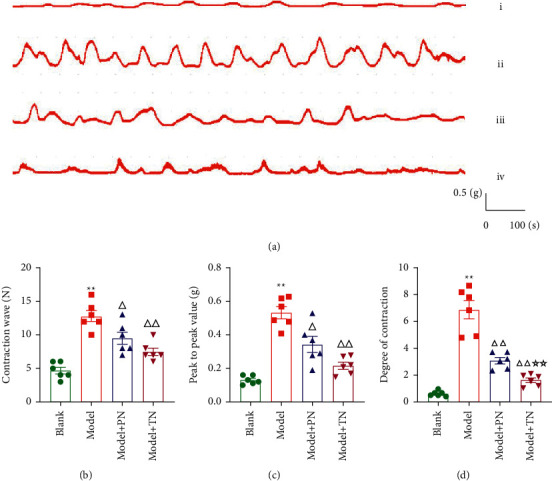
Acupuncture attenuated uterine contraction in CCD rats. (a) The representative uterine contraction curves of all groups. (i–iv) Blank group, Model group, Model + PN group, and Model + TN group, respectively. (b) The number of contraction wave. (c) The peak-to-peak value. (d) The degree of contraction. All data are expressed as the means ± SEM (*n* = 6 rats/group). (b) One-way ANOVA followed by the Bonferroni post hoc test was used. (c, d) The Tamhane T2 post hoc test was used. ^*∗∗*^*P* < 0.01 vs. Blank group; ^△△^*P* < 0.01 vs. Model group; ^△^*P* < 0.05 vs. Model group; ^☆☆^*P* < 0.01 vs. Model + PN group.

As presented in Figure 3, electrophysiological results showed that acupuncture attenuated uterine contractions in CCD rats (Figures 3(a)–3(d)). After model preparation, the number of uterine contraction wave, uterine peak-to-peak value, and uterine activity of model group were significantly increased (*P* < 0.01; Figures 3(b)–3(d)) compared with the blank group. After acupuncture, compared with the model group, the number of uterine contraction wave, the uterine peak-to-peak value, and uterine activity was significantly decreased (*P* < 0.05, *P* < 0.01; Figures 3(b)–3(d)) in two needling groups. Compared with the Model + PN group, uterine activity in the Model + TN group was significantly decreased (*P* < 0.01, Figure 3(d)).

The overall therapeutic effects of acupuncture with TN were superior to PN. Thus, we used TN at SP6 throughout the later experiments.

### 3.3. TN Reduced Eotaxin Levels and Attenuated the Expression of CCR3 in the Uterus

**Figure 4:**
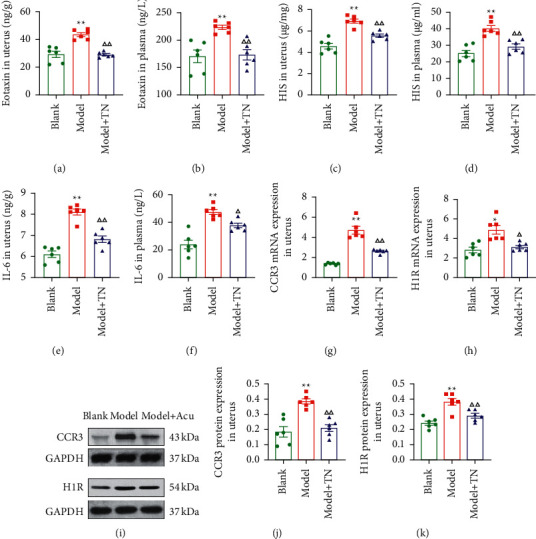
TN reduced eotaxin and histamine (HIS) levels and decreased the expression of CC chemokine receptor 3 (CCR3) and histamine H1 receptor (H1R) in CCD rats. The levels of eotaxin (a, b), HIS (c, d), and interleukin-6 (IL-6) (e, f) in uterus and plasma were detected by ELISA. The mRNA expression of CCR3 (g) and H1R (h) in the uterus was analyzed by RT-qPCR. (i–k) Representative blots (i), and protein expression levels of CCR3 (j) and H1R (k) in uterus. All data are expressed as the means ± SEM (*n* = 6 rats/group). (b–f, j, k) One-way ANOVA followed by the Bonferroni post hoc test was used. (a, g, h) The Tamhane T2 post hoc test was used. ^*∗∗*^*P* < 0.01 vs. Blank group; ^*∗*^*P* < 0.05 vs. Blank group; ^△△^*P* < 0.01 vs. Model group; ^△^*P* < 0.05 vs. Model group.

To determine whether TN could modulate eotaxin/CCR3 axis, we applied ELISA to detect the eotaxin level, RT-qPCR, and Western blot to analyze the expression of CCR3. Compared with the blank group, the eotaxin levels in uterus and plasma were significantly increased in the model group (*P* < 0.01; Figures 4(a) and 4(b)). In addition, we found that TN at SP6 attenuated this change (*P* < 0.01; Figures 4(a) and 4(b)). This attenuation was accompanied by decreased mRNA and protein expression of CCR3 (*P* < 0.01; Figures 4(g) and 4(j)).

### 3.4. TN Decreased Histamine Levels and the Expression of H1R in the Uterus

To investigate whether the HIS expression in uterus is associated with the eotaxin/CCR3 axis, we applied ELISA to detect the HIS level, RT-qPCR, and Western blot to analyze the expression of its receptor, H1R. The HIS level of model group was significantly increased compared with that of blank group in uterus and plasma (*P* < 0.01; Figures 4(c) and 4(d)). Furthermore, we found that TN at SP6 attenuated this change (*P* < 0.01; Figures 4(c) and 4(d)). This attenuation was accompanied by decreased mRNA and protein expression of H1R (*P* < 0.01, *P* < 0.05; Figures 4(h) and 4(k)).

### 3.5. TN Decreased IL-6 Levels in the Uterus and Plasma

To detect whether TN could modulate IL-6, we applied ELISA to identify IL-6 levels in uterus and plasma. The IL-6 levels of model group were significantly increased compared with those of blank group in both uterus and plasma (*P* < 0.01; Figures 4(e) and 4(f)). TN at SP6 decreased IL-6 levels in uterus and plasma (*P* < 0.01, *P* < 0.05; Figures 4(e) and 4(f)).

### 3.6. TN Attenuated the Activation of EOS and MCs in the Uterus

**Figure 5:**
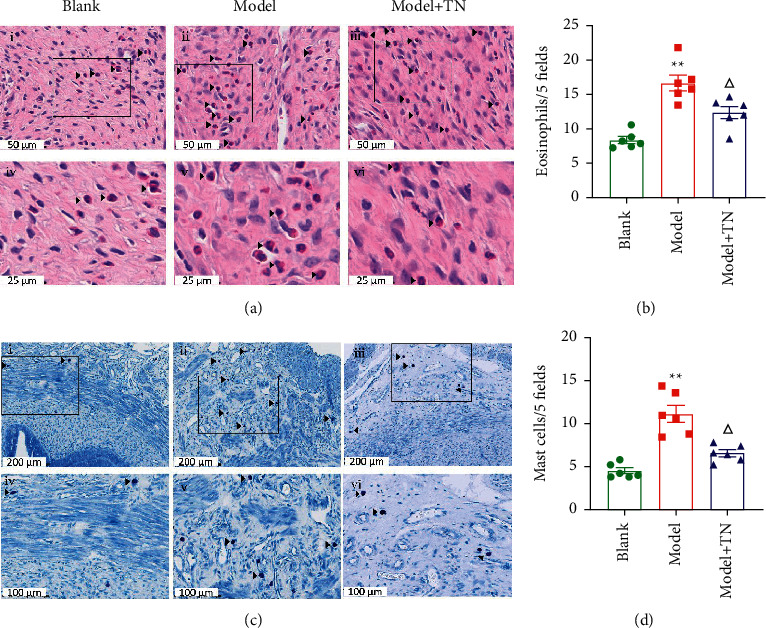
TN attenuated the expression of eosinophils (EOS) and mast cells (MCs) in the uterus of CCD rats. (a) The representative photomicrographs of EOS hematoxylin-eosin staining (HE) of all groups in the rat uterus. The regions represented by (i–iii) or (iv–vi) in (a) are photographs at 400x and 800x magnifications, respectively. (c) The representative photomicrographs of MCs' Toluidine Blue Staining (TB) of all groups in the rat uterus. The regions represented by (i–iii) or (iv–vi) in (c) are photographs at 200x and 400x magnifications, respectively. (b, d) A statistical histogram of the number of positive expression cells in each group in CCD rats. The arrows indicate EOS and MCs. All data are expressed as the means ± SEM (*n* = 6 rats/group). (b) One-way ANOVA followed by the Bonferroni post hoc test was used. (d) The Tamhane T2 post hoc test was used. ^*∗∗*^*P* < 0.01 vs. Blank group; ^△^*P* < 0.05 vs. Model group.

**Figure 6:**
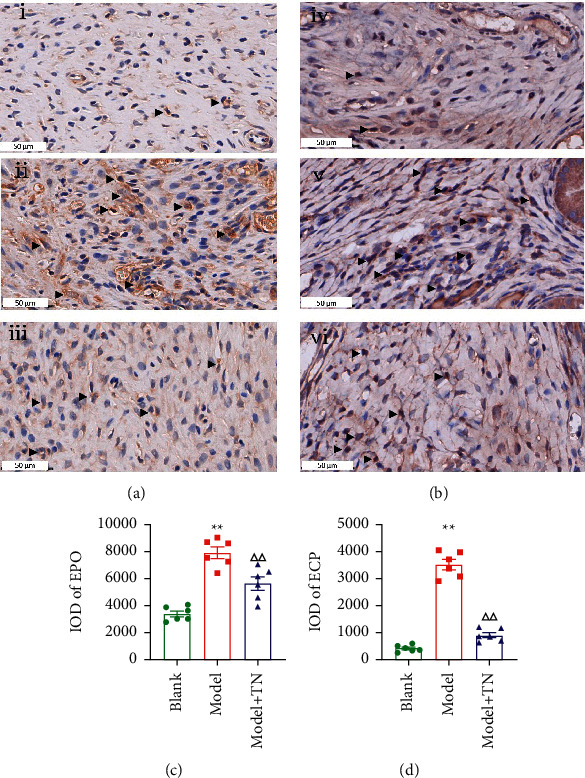
TN reduced the expression of eosinophil peroxidase (EPO) and eosinophil cationic protein (ECP) in the uterus of CCD rats. (a, b) The representative photomicrographs of EPO and ECP immunohistochemical staining of all groups in rat uterus. (c, d) Histogram of IOD value of EPO and ECP in the uterus of CCD rats in each group. (i and iv) Blank group; (ii and v) Model group; and (iii and vi) Model + TN group. The regions are photographs at 400x magnification. The arrows indicate a positive expression of EPO and ECP. All data are expressed as the means ± SEM (*n* = 6 rats/group). (c) One-way ANOVA followed by the Bonferroni post hoc test was used. (d) The Tamhane T2 post hoc test was used. ^*∗∗*^*P* < 0.01 vs. Blank group; ^△△^*P* < 0.01 vs. Model group.

To observe the association between eotaxin/CCR3 axis and HIS, we applied HE or TB, respectively, to analyze the main inflammatory cells, EOS and MC. As shown in Figure 5, compared with the model group, the Model + TN group attenuated the EOS and MC migration to uterus and their activation (Figures 5(a) and 5(c)). The number of EOS and MC in the Model + TN group decreased compared with the model group (*P* < 0.05; Figures 5(b) and 5(d)). Immunohistochemical staining results showed the positive expression of EPO (Figures 6(a)) and ECP (Figure 6(b)) of all groups in rat uterus. The IOD values of EPO and ECP in the Model + TN group decreased significantly compared with the model group (*P* < 0.01; Figures 6(c) and 6(d)).

### 3.7. CCR3 Agonist Blocked TN-Induced Analgesic Effect

**Figure 7:**
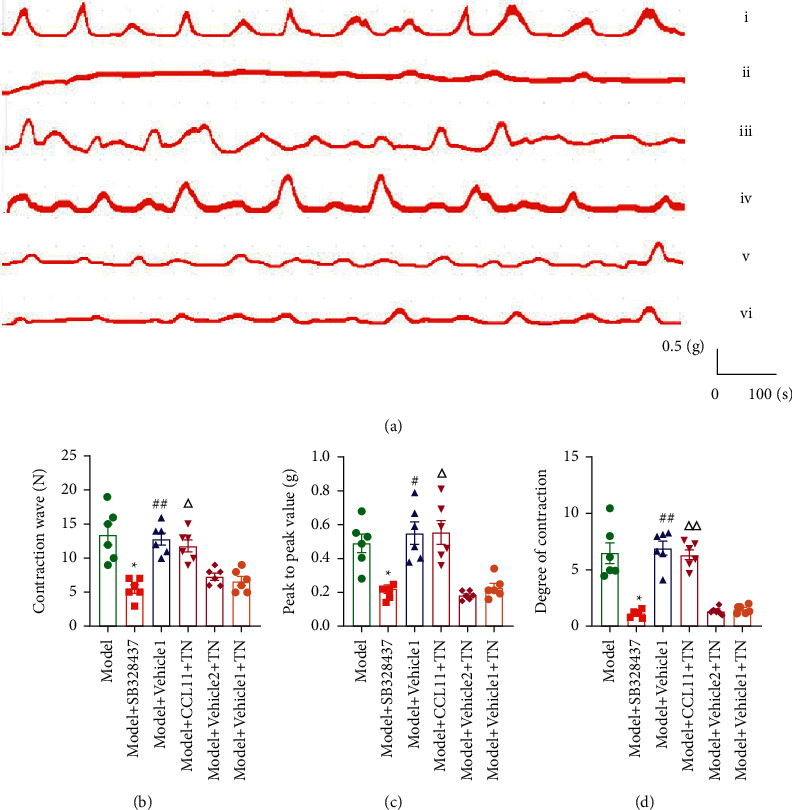
CCR3 agonist blocked the effect of TN against CCD-induced menstrual pain. (a) The representative uterine contraction curves of all groups. The representative images of the rat uterine activities. (i–vi) Model, Model + SB328437, Model + Vehicle1, Model + CCL11 + TN, Model + Vehicle2 + TN, and Model + Vehicle1 + TN. (b) The number of contraction wave. (c) The peak-to-peak value. (d) The degree of contraction. Data were analyzed by the Tamhane T2 post hoc test and expressed as means ± SEM (*n* = 6 rats/group). ^*∗*^*P* < 0.05 vs. Model group; ^△△^*P* < 0.01 vs. Model + Vehicle2 + TN; ^△^*P* < 0.05 vs. Model + Vehicle2 + TN; ^##^*P* < 0.01 vs. Model + Vehicle1 + TN; ^#^*P* < 0.05 vs. Model + Vehicle1 + TN.

TN at SP6 can relieve pain by attenuating the expression of eotaxin/CCR3 axis and EOS-MC activation. To further address whether pain-relief induced by TN was dependent on the activation of the eotaxin/CCR3 axis, we injected CCR3 antagonist SB328437 and agonist CCL11. Administration of SB328437 could mimic the effects of TN on uterine activity, and CCL11 blocked the effects of TN on uterine contractions in CCD rats (Figures 7(a)–7(d)). The number of contraction wave, uterine peak-to-peak value, and degree of contraction was significantly decreased compared with the model group after SB328437 application in the Model + SB328437 group (*P* < 0.05; Figures 7(b)–7(d)). However, after the application of CCL11 in the TN group, it markedly weakened TN-induced pain relief compared with the Model + Vehicle2 + TN group as manifested by the number of contraction wave, uterine peak-to-peak value, and degree of contraction (*P* < 0.01, *P* < 0.05; Figures 7(b)–7(d)). Compared with the Model + Vehicle1 + TN group, the number of contraction wave, uterine peak-to-peak value, and degree of contraction increased in the Model + Vehicle1 group (*P* < 0.01, *P* < 0.05; Figures 7(b)–7(d)). There was no significant difference in the number of contraction wave, peak-to-peak value, and degree of contraction between Model + Vehicle2 + TN and Model + Vehicle1 + TN groups (*P* > 0.05; Figures 7(b)–7(d)).

### 3.8. CCR3 Agonist Reversed the Decreased ECP Expression and HIS Level in the Uterus Induced by TN

**Figure 8:**
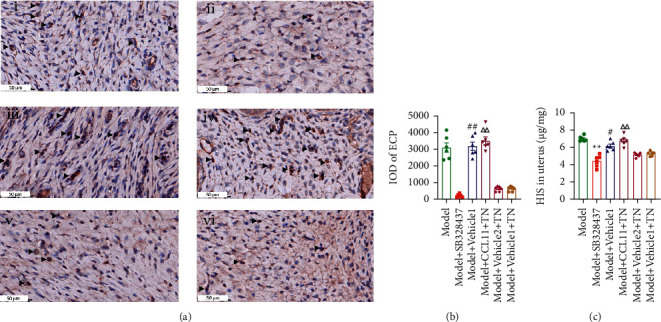
CCR3 agonist abolished the effect of TN against CCD-induced increased ECP expression and HIS level. (a) The representative photomicrographs of ECP immunohistochemical staining of all groups in the rat uterus. (i–vi) Model, Model + SB328437, Model + Vehicle1, Model + CCL11 + TN, Model + Vehicle2 + TN, and Model + Vehicle1 + TN. (b) Histogram of IOD value of ECP in the uterus of CCD rats in each group. (c) The level of HIS in uterus was detected by ELISA. The regions are photographs at 400x magnification. The arrows indicate a positive expression of ECP. All data are expressed as the means ± SEM (*n* = 6 rats/group). (b) The Tamhane T2 post hoc test was used. (c) One-way ANOVA followed by the Bonferroni post hoc test was used. ^*∗∗*^*P* < 0.01 vs. Model group; ^△△^*P* < 0.01 vs. Model + Viecle2 + TN; ^##^*P* < 0.01 vs. Model + Viecle1 + TN; ^#^*P* < 0.05 vs. Model + Viecle1 + TN.

After administration of SB328437, compared to the model group, the ECP expression and the HIS level were significantly decreased in the Model + SB328437 group (*P* < 0.01; Figures 8(a)–8(c)), which indicated that the antagonist of CCR3 could mimic the effects of TN. Compared to the Model + vehicle2 + TN group, the ECP expression and the HIS level were significantly increased in the Model + CCL11 + TN group (*P* < 0.01; Figures 8(a)–8(c)). Compared with the Model + Vehicle1 + TN group, the ECP expression and the HIS level increased in the Model + Vehicle1 group (*P* < 0.01, *P* < 0.05; Figures 8(a)–8(c)). There was no significant difference in the ECP expression and the HIS level between Model + Vehicle2 + TN and Model + Vehicle1 + TN groups (*P* > 0.05; Figures 8(a)–8(c)).

## 4. Discussion

In this study, acupuncture significantly relieved menstrual pain in CCD rats. These positive therapeutic effects may be achieved through the eotaxin/CCR3 axis, as manifested by weakening EOS-MC activation and restraining release of HIS. Moreover, the CCR3 agonist CCL11 significantly abolished a TN-induced analgesic effect and related benefits, while CCR3 antagonist SB328437 could mimic the analgesic effect, supporting this contention.

PD is a common gynecological disorder and the pain was attributed to increased uterine contractility [38, 39]. As an economical and practical therapy, acupuncture can be applied in relieving menstrual pain [18–20]. PD with cold congealing syndrome in traditional Chinese medicine (TCM) is the most common pattern [29]. Clinical and experimental studies indicated that alterations in a needling technique have an impact on therapeutic outcomes [40–42]. Although SP6 has been affirmed as one of the most commonly used acupoints for PD [27–29], which needling technique is the most effective method remains unclear. In order to reduce the interference of other acupoints and better observe the influence of different needling techniques on acupoints, a solo acupoint SP6 was used in the present study. To investigate the influence of needling techniques on SP6's analgesic effect is crucial for acupuncture clinical practice. TN, almost subcutaneously inserting the needle, is believed to be an adaptive needling technique for cold congealing syndrome according to the classic TCM theory. Our results suggested that increased uterine contraction was ameliorated by acupuncture treatment. Moreover, the overall therapeutic effect of TN was superior over PN.

The leukocytic invasion and subsequent production of inflammatory mediators are observed during menstruation; hence, the menstruation could be considered as an inflammatory event [6]. When dysmenorrhea occurs, the inflammatory response of the uterus increases [43]. As an important aspect of promoting inflammation and causing tissue damage, EOS plays a major role in the pathophysiology of PD. The involvement of eosinophils in the female reproductive tract has been reported since the 1960s [44]. Also, in recent years, the role of EOS in the reproductive system has attracted a lot of attention [7, 8, 15, 36]. Studies found that EOS became evident prior to and during menstruation [7–9]. Chemokines and their cognate receptors play an important role in the control of leukocyte chemotaxis. Eotaxin, a member of the C-C chemokine family, is a specific chemotactic agent for EOS into the inflammatory uterus [45]. Eotaxin chemokines are grouped into 3 subtypes of eotaxin-1/CCL11, eotaxin-2/CCL24, and eotaxin-3/CCL26 [46], which are structurally different but functionally similar, selectively activating EOS by binding to a common receptor, CC chemokine receptor 3 (CCR3) [45]. And it is reported that CCL11 is the most closely associated with the uterus [47]. Eosinophil peroxidase (EPO) is utilized to assess the migration and activation of EOS. In this study, the expression of eotaxin, EOS, and EPO was increased in CCD rats, and acupuncture significantly reversed this change. Interleukin 6(IL-6) is a multifunctional proinflammatory cytokine and has multidirectional effects on both innate and acquired immune system cells. Studies have shown that plasma IL-6 concentration is related to the menstrual cycle [48], and IL-6 can promote the synthesis or release of prostaglandins [49]. The plasma IL-6 concentration increased on the first day of menstruation in women with PD [50], leading to excessive contraction of uterine muscles and reduced blood flow, ultimately ischemic pain. Currently, studies have proved that it is an effective way to treat dysmenorrhea by regulating IL-6 concentration [51]. In our study, we detected the levels of IL-6 in plasma and uterus and found that acupuncture could reduce the IL-6 levels, further verifying the anti-inflammatory effect of acupuncture.

It was reported that MCs and EOS were found at close range [52]. EOS have the capacity to modulate MC functions, such as producing ECP to stimulate HIS release from MCs [53]. In this study, the expression of ECP, MC, and HIS was increased in CCD rats, and the increase was reversed by acupuncture. The activation of inflammatory cells leads to the release of HIS, which is an important cause of dysmenorrhea. It was reported that HIS can promote the production of estradiol and PGF2*α* when combined with H1R, which in turn promotes uterine contractility and exacerbates pain [16]. In addition, the contraction of rat vascular smooth muscle was induced by HIS through H1R [54]. A study showed that both MCs' stabilizer group and H1R antagonist group could inhibit uterine contraction [55]. Furthermore, H1R gene knockout mice had lower pain sensitivity and higher pain threshold than normal mice [56]. Similarly, our research indicated that rats showed obvious writhing response and increased uterine contractility after modeling along with the increased HIS level and H1R expression, while pain behaviors and uterine contractility were relieved accompanied by decreased HIS level and H1R expression after acupuncture.

Although eotaxin is important in dysmenorrhea, the mechanism remains unclear. CC chemokine receptor 3 (CCR3), a 7-transmembrane-domain G protein-coupled receptor, is expressed selectively in EOS, making it an excellent candidate for an eotaxin receptor [45, 57]. Previous researches showed that the eotaxin/CCR3 axis is a key regulatory pathway for EOS recruitment and migration to inflammatory tissues [45, 58, 59]. Allergen-induced airway EOS recruitment was almost eliminated in CCR3-deficient and eotaxin knockout mice (70%) [60]. Simultaneously, another research indicated that a CCR3 antagonist impaired EOS migration in the rat uterus [36]. What's more, acupuncture showed positive results of relieving asthma and allergic rhinitis via reducing eotaxin secretion [24, 25]. In our study, the expression of CCR3 was significantly increased in CCD rats, but TN reversed the change. Moreover, our present study also found that the effects of TN against CCD-induced menstrual pain, increased ECP expression, and HIS level could be blocked by CCR3 agonist CCL11, which further provided a strong support for our conclusion that eotaxin exerted its effect in menstrual pain at least partly through the activation of CCR3. To prove it further, CCR3 antagonist SB328437 could simulate the effect of TN, significantly ameliorating the uterine contraction and reducing ECP expression and HIS level in CCD rats.

There are some limitations in our study. First, the local signal amplification and transmission caused by TN and PN at SP6 as well as the regulation of central neuroendocrine system on target organ and related molecules should be further improved. Second, changes of classical inflammatory factors in uterine inflammatory microenvironment induced by acupuncture, especially those related to EOS and MC, deserve further observation.

## 5. Conclusion

This study reported a preferable effect of acupuncture at SP6 with TN on pain relief in CCD rats, which was partly achieved by weakening EOS-MC activation and HIS release via inactivation of eotaxin and CCR3. Accordingly, our data support that acupuncture as a potentially promising therapeutic approach for the treatment of primary dysmenorrhea, meanwhile, indicating the importance of performing appropriate needling technique.

## Figures and Tables

**Table 1:** The primers used for RT-qPCR analysis.

Name		Sequences (5′-3′)
*β*-Actin	Forward primer	5′-GCACCATGAAGATCAAGATCAT
	Reverse primer	3′-TAACAGTCCGCCTAGAAGCATT

CCR3	Forward primer	5′- GTCTTGCAGTATTGGCAGCAT
	Reverse primer	3′-TTCTTCGCCCTCTGGATAGC

H1R	Forward primer	5′- TGTGTGAGGGGAACAGGACA
	Reverse primer	3′- ACAGCACCAGCAGGTTGAGG

## Data Availability

All data in the current study are available from the corresponding authors on reasonable request.
